# Systematic re-evaluation of the bis(2-hydroxyethyl)disulfide (HEDS) assay reveals an alternative mechanism and activity of glutaredoxins†
†Electronic supplementary information (ESI) available: Alternative evaluation of the HEDS assay with ScGrx7 (Fig. S1), HEDS and GSSEtOH assay with PfGrx (Fig. S2), enzyme parameters for the GSSEtOH assay with ScGrx7 (Table S1) and calculations of estimated reaction velocities and substrate concentrations (Tables S2–4). See DOI: 10.1039/c5sc01051a



**DOI:** 10.1039/c5sc01051a

**Published:** 2015-05-19

**Authors:** Patricia Begas, Verena Staudacher, Marcel Deponte

**Affiliations:** a Department of Parasitology , Ruprecht-Karls University , D-69120 Heidelberg , Germany . Email: marcel.deponte@gmx.de

## Abstract

The reduction of bis(2-hydroxyethyl)disulfide (HEDS) by reduced glutathione (GSH) is the most commonly used assay to analyze the presence and properties of enzymatically active glutaredoxins (Grx), a family of central redox proteins in eukaryotes and glutathione-utilizing prokaryotes. Enzymatically active Grx usually prefer glutathionylated disulfide substrates. These are converted *via* a ping-pong mechanism. Sequential kinetic patterns for the HEDS assay have therefore been puzzling since 1991. Here we established a novel assay and used the model enzyme ScGrx7 from yeast and PfGrx from *Plasmodium falciparum* to test several possible causes for the sequential kinetics such as pre-enzymatic GSH depletion, simultaneous binding of a glutathionylated substrate and GSH, as well as substrate or product inhibition. Furthermore, we analyzed the non-enzymatic reaction between HEDS and GSH by HPLC and mass spectrometry suggesting that such a reaction is too slow to explain high Grx activities in the assay. The most plausible interpretation of our results is a direct Grx-catalyzed reduction of HEDS. Physiological implications of this alternative mechanism and of the Grx-catalyzed reduction of non-glutathione disulfide substrates are discussed.

## Abstract 2

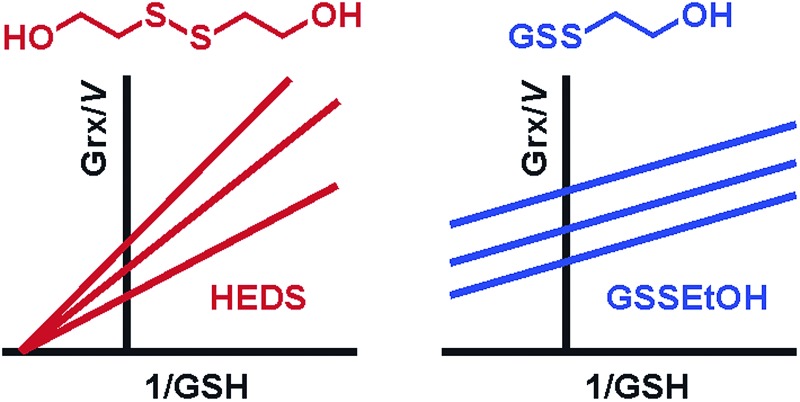
The sequential kinetic patterns of mono- and dithiol glutaredoxins in the HEDS assay reflect an alternative enzymatic mechanism for the glutathione-dependent reduction of disulfide substrates.

## Introduction

**Fig. 1:**
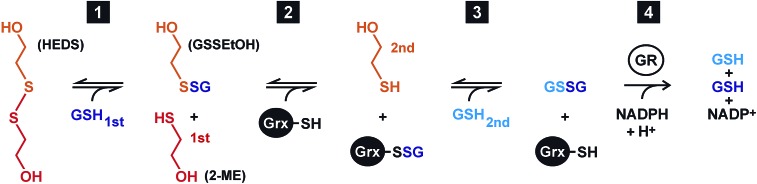
Mechanistic model of the HEDS assay. Reaction 1 between HEDS and GSH is thought to be Grx-independent, whereas the reduction of GSSEtOH yielding GSSG is catalyzed by Grx *via* a ping-pong monothiol mechanism (reactions 2 and 3). The reduction of GSSG is subsequently monitored by the GR-catalyzed consumption of NADPH (reaction 4).

In 1968, Nagai and Black established the bis(2-hydroxyethyl)disulfide (HEDS) assay for the analysis of a purified GSH:disulfide oxidoreductase from yeast.^1^ Since then, the assay became the most commonly used method to determine the presence, activity and enzyme kinetic parameters of glutaredoxins (Grx) from all kinds of organisms and sources.^2–6^ The HEDS assay has two major advantages. First, HEDS is a rather inexpensive commercially available disulfide substrate. Second, the formation of glutathione disulfide (GSSG) can be monitored spectrophotometrically in a robust coupled assay owing to the consumption of NADPH by glutathione reductase (GR) (Fig. 1). It is therefore surprising that the exact mechanism of the analyzed reaction is still unclear,^2,4,7–9^ in particular, taking into account that such a mechanism might reveal fundamental insights with regard to the poorly understood structure–function relationships of enzymatically active and inactive Grx-isoforms.^6,8^

According to a mechanistic model by Gravina and Mieyal^7^ as well as Bushweller *et al.*,^10^ HEDS initially reacts non-enzymatically with GSH (reaction 1 in Fig. 1). The obtained mixed disulfide between GSH and 2-mercaptoethanol (2-ME), GSSEtOH, is considered to be the actual substrate of Grx in the assay. The active site of Grx contains a conserved cysteine residue in its thiolate form. During the oxidative half-reaction, the thiolate attacks GSSEtOH and becomes glutathionylated (reaction 2). The reduced enzyme is subsequently regenerated during the reductive half-reaction of the ping-pong mechanism with the help of a second GSH molecule yielding GSSG (reaction 3).^7,10^ Many Grx have a second cysteine residue in a CxxC-motif and/or another cysteine in a GGC-motif in proximity to the active site cysteine(s). These residues allow the formation of alternative intra- and intermolecular disulfide bonds.^6,11–14^ Mutational analyses in the early 1990s revealed that the second cysteine residue of the CxxC-motif of so-called dithiol Grx is dispensable for the enzymatic activity in the HEDS assay.^10,11,15^ This finding was later confirmed for numerous Grx-isoforms and glutathionylated substrates including l-cysteine-glutathione disulfide (GSSCys).^4,14–19^ One exception is ScGrx8, an unusual dithiol Grx from yeast with a low enzymatic activity that is lost when the second cysteine residue of the CxxC-motif is replaced.^8^ Of note, monothiol Grx-isoforms, which have a CxxS-motif, are usually inactive in the HEDS assay.^12,13,20–22^ As reviewed recently,^6^ plausible explanations for the enzymatic inactivity of Grx-isoforms might be structural peculiarities that result in an absent activation of the second GSH molecule as a nucleophile, poor leaving group properties of the active site cysteine thiolate, or geometric constraints such as trapped enzyme conformations in the absence of a so-called resolving cysteine residue. To date, yeast ScGrx6 and ScGrx7, which are found in the endoplasmic reticulum and Golgi,^23,24^ are the only monothiol Grx-isoforms with significant activity in the HEDS assay.^4,8,23–25^ Both proteins form non-covalent dimers, possess a single cysteine residue per subunit and share structural features with dithiol Grx-isoforms.^4,25^ The overall kinetics of ScGrx7 are neither complicated by the formation of intramolecular disulfide bonds nor by iron–sulfur cluster binding. Hence, ScGrx7 is an excellent model enzyme to address mechanistic questions.^4,8^

The most puzzling aspect about the HEDS assay are the sequential kinetic patterns for mammalian dithiol Grx and monothiol ScGrx7 with common intersection points in Lineweaver–Burk plots which are not in accordance with a simple ping-pong mechanism.^2,4^ Potential reasons for the sequential kinetic patterns in the HEDS assay are:^2,4,6–9^ (i) the actual concentration of GSH in the assay is undefined because of the unknown position of the equilibrium of reaction 1. Deviations from the expected ping-pong patterns might therefore be due to decreased concentrations of available GSH in the assay. (ii) Kinetics with GSSCys and GSH previously revealed ping-pong patterns for mammalian dithiol Grx and ScGrx7.^4,7,19^ However, GSSEtOH is smaller and lacks the charges of the cysteine moiety of GSSCys. A sequential pattern might therefore reflect the simultaneous binding of GSSEtOH and GSH at two alternative binding sites. (iii) Reactions 1 and 2 each yield one molecule 2-ME, which might cause sequential patterns owing to product inhibition. Alternatively, HEDS or GSH might cause substrate inhibition. (iv) When the assay is started with HEDS, a lag phase is observed,^2,4,8^ and a non-enzymatic formation of GSSEtOH in reaction 1 could therefore be rate-limiting.^7^ (v) Last but not least, HEDS and GSH might be actually converted by Grx *via* a sequential mechanism. Here we addressed aspects (i–v) by comparing the kinetics of the HEDS assay with the kinetics of a novel GSSEtOH assay and the kinetics of reaction 1 without enzymes. Our data support a direct Grx-catalyzed reduction of HEDS reflecting an alternative Grx activity with a non-glutathione disulfide substrate.

## Results

### Effect of the estimated GSH concentration

The formation of each molecule GSSEtOH is coupled to the consumption of one molecule GSH (Fig. 1). In order to determine the influence of incorrect estimations regarding the concentration of available GSH in the HEDS assay, we performed alternative evaluations of the steady-state kinetics for ScGrx7 (Fig. S1†). First, we assumed that different percentages of HEDS had reacted with GSH during the 2 min pre-incubation step before the enzyme was added. The GSH concentrations were corrected and plotted accordingly (Fig. S1A†). Best fits of kinetic data, as reflected by the *r* ^2^ values from the non-linear and linear regression analyses, were obtained for the uncorrected initial GSH concentrations. Under these conditions the data sets revealed a rather constant *K*appm value for GSH around 1.5 mM (as exemplified by a common intersection point at the *x*-axis in Lineweaver–Burk plots) in accordance with previous measurements.^4^ Similar *r* ^2^ values and patterns were obtained for the assumption that about 10% of HEDS had reacted, whereas models with higher percentages resulted in poor fits (Fig. S1A†). Next, we assumed different hypothetical equilibrium constants for reaction 1 from Fig. 1 and calculated the concentration of free GSH using eqn (1) as described in the experimental section. Models for hypothetical apparent *K* (*K* ^app^) values <2, indicating rather efficient GSSEtOH formation in the assay, resulted in poor fits in contrast to models for less efficient GSSEtOH formation with *K* ^app^ values ≥2 (Fig. S1B†). Increasing the hypothetical equilibrium constant above 10^2^ neither yielded improved fits nor altered the sequential kinetic patterns. In an independent approach we experimentally estimated the *K* ^app^ value by HPLC at a variety of substrate concentrations (*n* = 18). Quantification of the HPLC peaks for HEDS and 2-ME after 2 min pre-incubation without ScGrx7 were in good agreement with the models in Fig. S1† and yielded *K* ^app^ values of 8.2 ± 4.1 and 9.8 ± 5.0, respectively. Hence, regardless of the experimental approach, the data altogether indicate that only little HEDS and GSH had been consumed under the chosen assay conditions. In summary, incorrect estimations regarding the net concentration of GSH in the HEDS assay are not the cause for the sequential kinetic patterns.

### Establishment of a GSSEtOH assay

**Fig. 2:**
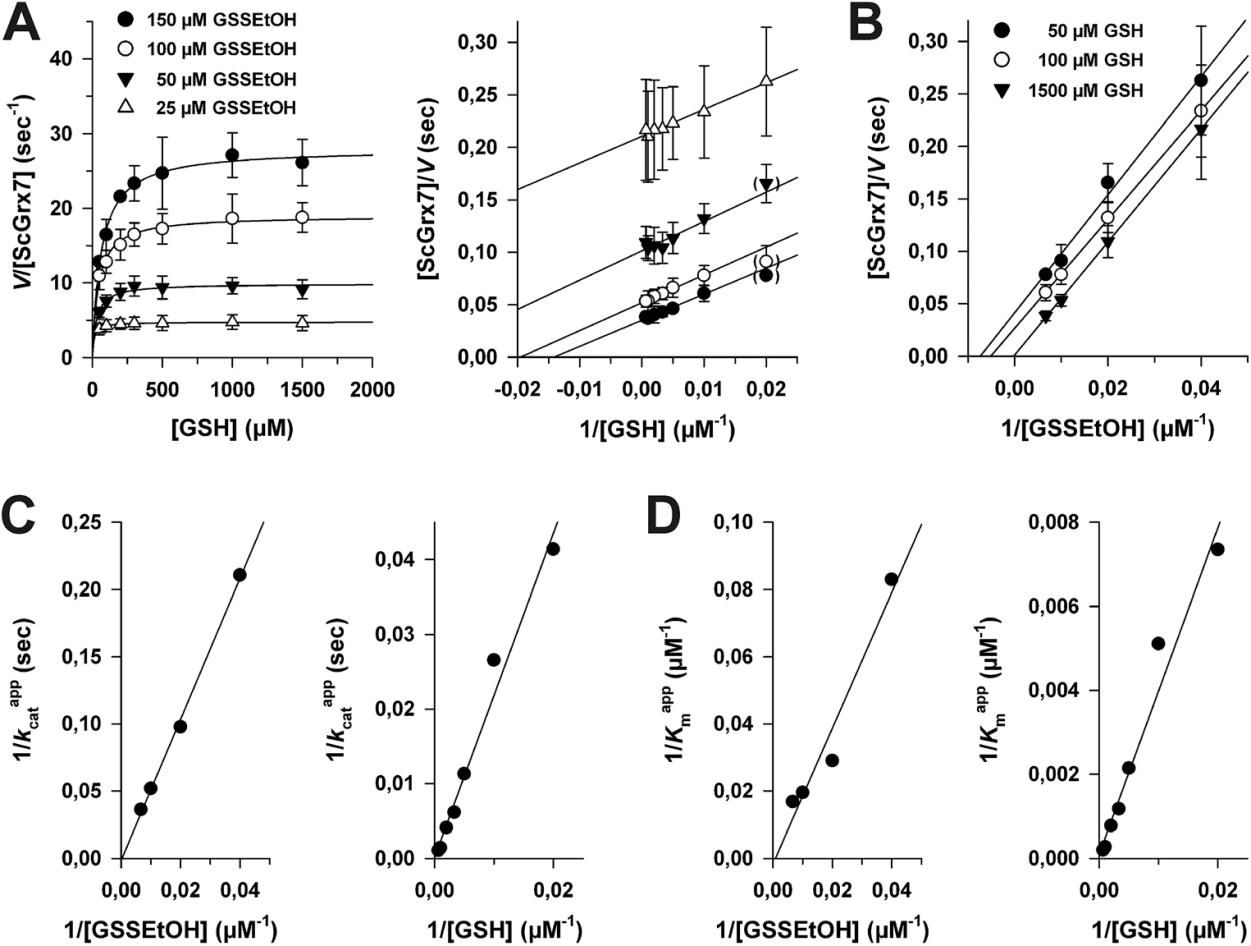
Steady-state kinetics for ScGrx7 in the GSSEtOH assay. (A) Michaelis–Menten and Lineweaver–Burk plots of the GSH-dependent reaction velocity at different initial concentrations of GSSEtOH. Data points at low substrate concentrations in brackets were omitted for the linear regression analysis. (B) Lineweaver–Burk plots of the GSSEtOH-dependent reaction velocity at different initial concentrations of GSH. Apparent kinetic constants are listed in Table S1.† Values for each data point in panels A and B were averaged from three independent experiments. (C) and (D) Secondary plots of the kinetic constants and extrapolation of the true *k*_cat_ and *K*_m_ values.

In order to discriminate whether a reaction between HEDS and GSH causes the sequential kinetics, or whether GSSEtOH and GSH simultaneously bind to ScGrx7, we synthesized and purified GSSEtOH as described in the experimental section. The reduction of GSSEtOH by GSH was subsequently analyzed in an analogous coupled photometric assay (Fig. 2). In contrast to the sequential patterns for the HEDS assay (Fig. S1†), the kinetics with purified GSSEtOH yielded parallel lines in Lineweaver–Burk plots (Fig. 2A and B), which is indicative of a ping-pong mechanism. Of note, the *K*appm values for GSH at the chosen assay conditions were below 100 μM suggesting a high affinity of ScGrx7 in the presence of low disulfide substrate concentrations. Furthermore, when the GSH concentration was kept constant at 50 or 100 μM, the *K*appm value for GSSEtOH was roughly four times higher than the *K*appm value for GSH at 50 or 100 μM GSSEtOH (Table S1†). This suggests that either the 2-ME moiety of GSSEtOH increases the *K*appm or that the binding sites for GSH and GSSEtOH differ (see also ref. 6 and 8). An evaluation of the apparent kinetic parameters in secondary plots revealed intersection points close to the origin of the graphs (Fig. 2C and D) and the true *k*_cat_ and *K*_m_ values of ScGrx7 tended to be infinite in contrast to previous preliminary estimations.^4^

Next, we tested whether a switch from sequential to ping-pong patterns is also observed for other enzymes including dithiol Grx. We therefore used an alternative system consisting of the recombinant *Plasmodium falciparum* enzymes PfGrx and PfGR.^14^ In accordance with the results for ScGrx7, HEDS assays with PfGrx yielded sequential kinetic patterns whereas ping-pong patterns were detected for GSSEtOH (Fig. S2†). Please note that PfGrx has three cysteines (residues 29 and 32 in a typical CPYC-motif and residue 88 in a GGC-motif).^14^ Hence, the assay-dependent switch of the kinetic patterns can be observed for monothiol and dithiol Grx regardless of the presence or absence of additional cysteine residues. In summary, the Grx-dependent reduction of GSSEtOH is catalyzed *via* a ping-pong mechanism. We therefore exclude simultaneous binding of GSSEtOH and GSH as a cause for the sequential patterns in the HEDS assay.

### The influence of 2-ME

**Fig. 3:**
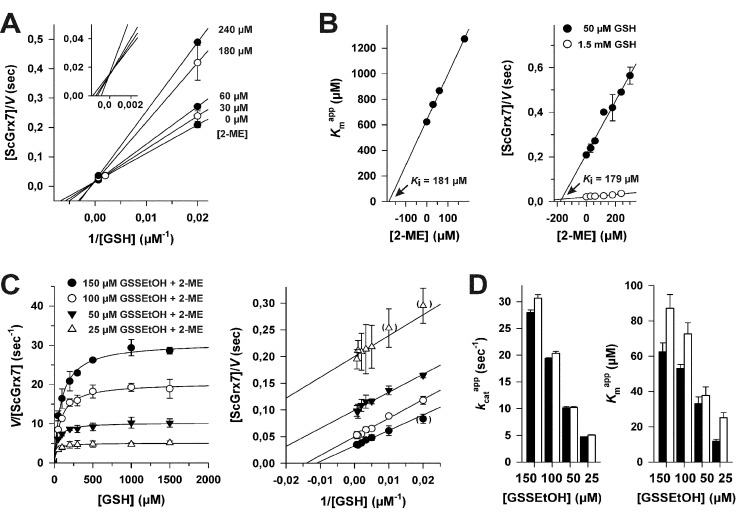
Influence of 2-ME on ScGrx7 in the HEDS and GSSEtOH assay. (A) Product inhibition patterns for 2-ME in the HEDS assay according to Lineweaver–Burk theory. (B) The *K*_i_ value for 2-ME in the presence of 0.74 mM HEDS was determined by replotting the *K*appm values for GSH from panel A *versus* the inhibitor concentration (left side). Alternatively, the *K*_i_ was determined from the intersection point in a Dixon plot (right side). (C) GSH-dependency of the reaction velocity in the GSSEtOH assay at different initial concentrations of GSSEtOH in the presence of equimolar amounts of 2-ME. Data points at low substrate concentrations in brackets were omitted for the linear regression analysis. (D) Comparison of *k*appcat and *K*appm(GSH) values from GSSEtOH assays in the absence (closed bars) or presence (open bars) of equimolar amounts of 2-ME. Values for each data point in panels A–C were averaged from two independent experiments.

To determine the influence of 2-ME from reaction 1 on the overall kinetics, we performed product inhibition studies in the HEDS and GSSEtOH assay (Fig. 3). When 2-ME was added to the HEDS assay before the reaction was started with ScGrx7, a common intersection point at the *y*-axis of the Lineweaver–Burk plot was observed, which is indicative of a competitive inhibition (Fig. 3A). The *k*appcat for 0.74 mM HEDS at different GSH and 2-ME concentrations was 66 ± 3 s^–1^, which is slightly higher than previously described.^4^ Re-plots of the *K*appm values for GSH *versus* the concentration of 2-ME as well as Dixon plots both revealed an inhibition constant *K*_i_ for 2-ME of 0.18 mM (Fig. 3B). Taking into account the results from Fig. S1† and the estimated *K* ^app^ value from HPLC analyses, the 2-ME concentration in the standard HEDS assay was significantly lower than the *K*_i_ value. Nevertheless, we also checked whether the ping-pong patterns in Fig. 2 can be converted to sequential patterns by adding equimolar amounts of 2-ME and disulfide substrate to the GSSEtOH assay. Starting the reaction with the disulfide substrate yielded similar ping-pong patterns as in Fig. 2 (Fig. 3C). The *k*appcat values in the presence or absence of 2-ME were almost identical, and the *K*appm(GSH) values in the presence of 2-ME increased moderately at all tested GSSEtOH concentrations (Fig. 3D). In summary, even though 2-ME is a competitive inhibitor of ScGrx7, the amount of 2-ME that is liberated under the chosen assay conditions is not sufficient to convert the ping-pong patterns of the GSSEtOH assay to the sequential patterns of the HEDS assay. Hence, 2-ME is not the cause for the sequential patterns in the HEDS assay.

### Characterization of reaction 1

**Fig. 4:**
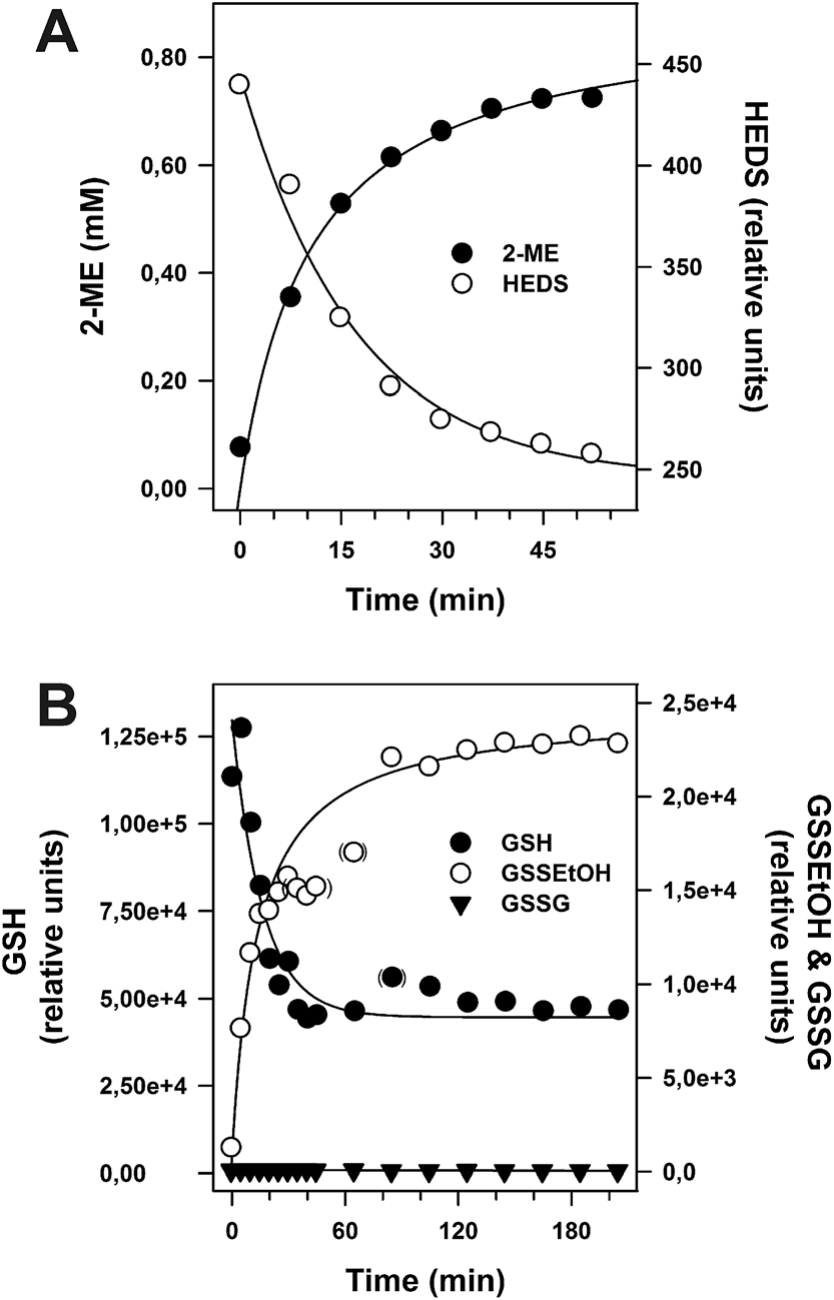
Non-enzymatic reaction between 1.2 mM HEDS and 1.2 mM GSH. (A) The time-dependent formation of 2-ME and consumption of HEDS were analyzed by HPLC. Calibration of the 2-ME signal and subsequent regression analysis of the obtained concentrations over time revealed that approx. 70% of the substrate was converted to product at equilibrium. A concentration of 0.6 mM 2-ME was reached after 22 min. (B) The time-dependent consumption of GSH and formation of GSSEtOH and GSSG were analyzed by mass spectrometry. Semi-quantitative analysis of the GSH peak areas revealed a consumption of approx. 65% of substrate at equilibrium.

A central problem of the standard HEDS assay is that the postulated non-enzymatic formation of GSSEtOH is monitored indirectly by an enzymatic detection system (Fig. 1). In order to monitor reaction 1 without NADPH, GR and Grx, we directly analyzed the non-enzymatic consumption of HEDS/GSH and the formation of 2-ME/GSSEtOH in assay buffer by HPLC (Fig. 4A) and mass spectrometry (Fig. 4B). Both approaches revealed an equilibration time ≥60 min and a consumption of approx. 70% of the substrates at equilibrium. GSSEtOH and GSH did not appear to react to a significant extent under the chosen conditions as mass spectrometry showed no GSSG formation. We therefore estimated an equilibrium constant *K* around 0.18, which is more than one order of magnitude lower than the *K* ^app^ values determined after two minutes pre-incubation. After 22 minutes, 50% of 1.2 mM HEDS had reacted to 0.6 mM 2-ME (Fig. 4A). A similar *t*_1/2_ value was extrapolated for the formation of GSSEtOH from 1.2 mM GSH. Based on a kinetic law with *v* = *k*_2_[GSH][HEDS] and the correlation *t*_1/2_^–1^ = *k*_2_[HEDS]_i_ for [GSH] = [HEDS], we estimated a second order rate constant *k*obs2 of 0.63 M^–1^ s^–1^. This rate constant was used to extrapolate the concentrations of GSH and HEDS after two minutes pre-incubation (Table S2†). The data are in good agreement with Fig. S1† suggesting that less than 10% of GSH and HEDS were consumed for the analyzed substrate concentrations. However, in accordance with experimental observations, Table S2† also suggests that it is problematic to use GSH or HEDS concentrations of more than 1.5 mM because this will lead to a significant consumption of substrate during pre-incubation and an overestimation of the remaining substrate concentration in the HEDS assay. The effect should furthermore increase with longer pre-incubation periods (Table S3†).

**Fig. 5:**
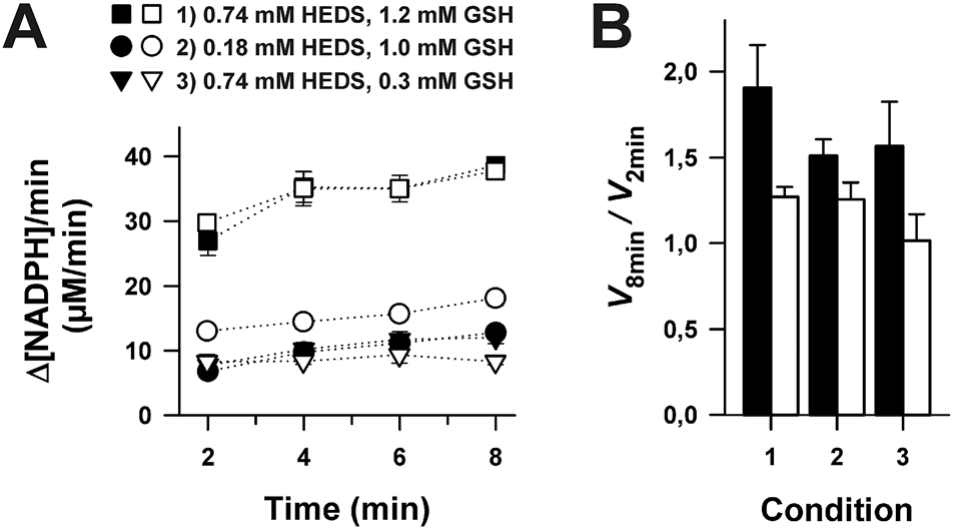
Relevance of the pre-incubation period for the enzymatic activity in the HEDS assay. (A) Correlation between the reaction velocity and the length of the pre-incubation period in the absence of ScGrx7. All assays were performed with 12.5 nM ScGrx7 (closed symbols) or PfGrx^C32S/C88S^ (open symbols) at the indicated conditions 1–3. (B) Ratio between the measured reaction velocities from panel A after 2 and 8 min pre-incubation. Values for each data point were averaged from three measurements.

Fig. 4 shows that the concentration of GSSEtOH changed significantly during the pre-incubation period. Within the first few minutes, the correlation between GSSEtOH formation and time was almost linear (Fig. 4B). A four-fold prolongation of the pre-incubation period from 2 to 8 minutes should therefore result in an almost four-fold increase of the amount of available GSSEtOH in the HEDS assay (Table S3†). This is expected to significantly alter the enzyme kinetics (because the enzyme should be far from being saturated according to the *K*appm(GSSEtOH) values from Fig. 2B and D). However, the reaction velocity only increased about 1.5 to 1.9-fold when the pre-incubation period was quadrupled (Fig. 5). The effect appears to depend on the substrate concentration as well as the ScGrx7 and/or GR enzyme preparation since previous measurements showed no significant change after 10 min pre-incubation.^4^ We therefore also analyzed our PfGrx/PfGR system using 1 U mL^–1^ PfGR and the fully functional monothiol mutant PfGrx^C32S/C88S^, which allows a direct comparison with monothiol ScGrx7 because of absent side reactions.^14^ This system was quite robust with no time-dependent change of reaction velocity at 0.74 mM HEDS and 0.3 mM GSH and a just 1.2-fold increase of reaction velocity at 0.74 mM HEDS and 1.2 mM GSH (Fig. 5B). GR was not rate-limiting because addition of 2 instead of 1 U mL^–1^ to the assay yielded an identical activity in accordance with previous measurements.^4,8,14^ Thus, the relevance of the pre-incubation period depends on the investigated enzyme system and pre-incubation is far less relevant for the measured activity of ScGrx7 and PfGrx^C32S/C88S^ than expected. Both aspects argue against a non-enzymatic formation of GSSEtOH as a prerequisite for the measured activity. To further support this interpretation, we compared the reaction velocities at the calculated GSSEtOH concentrations in the HEDS assay from Fig. S1† and the measured activities at similar substrate concentrations in the GSSEtOH assay from Fig. 2. Assuming a non-enzymatic reaction 1, the measured activities in the HEDS assay were about 3–6 times too high to be in accordance with the calculated GSSEtOH concentrations (Table S4†). In summary, a Grx-catalyzed reaction between HEDS and GSH is the most plausible explanation not only for the high activity of ScGrx7 and PfGrx^C32S/C88S^ in the assay but also for the system-dependent (ir-)relevance of the length of the pre-incubation period and the sequential kinetic patterns.

## Discussion

The underlying mechanism for the sequential kinetic patterns in the HEDS assay has been a matter of debate for more than two decades. In 1991, Mieyal *et al.* originally proposed a hypothetical sequential mechanism for GSH and HEDS for which “bond-breaking/making events leading to release of (2-ME) would not occur until after (GSH) was bound”.^2^ This mechanism involved the formation of a postulated Grx-SSEtOH intermediate that would be attacked by GSH. The sequential model was later questioned because subsequent studies with glutathionylated disulfide substrates such as bovine serum albumin or GSSCys yielded ping-pong patterns for a variety of enzymes. A Grx-SSG intermediate was therefore suggested to be also formed in the HEDS assay.^4,7,19,26,27^ Since Grx preferentially recognize glutathionylated substrates and inefficiently reduce model protein substrates such as cysteinylated serum albumin, hemoglobin or papain, the sequential patterns for the HEDS assay were attributed to a rate-limiting, non-enzymatic GSSEtOH formation as depicted in Fig. 1.^7^ However, some Grx are able to efficiently reduce selected non-glutathionylated protein disulfides such as bacterial ribonucleotide reductase or 3′-phosphoadenosine 5′-phosphosulfate reductase,^5,6,10,28,29^ and it is therefore problematic to completely exclude non-glutathionylated substrates, in particular, when they are small and can easily access the active site. A quite rapid reduction of a number of small non-glutathione substrates such as cystine has actually been reported in previous indirect assays.^1,2^ Furthermore, Grx and glutathione transferases (GST) sometimes have overlapping enzymatic activities because of interchanged catalytic and substrate binding residues,^6^ and a GST-like conjugase activity has been reported for yeast Grx.^25,30^ Hence, there are three potentially competing reactions for the reduction of HEDS that could be rate-limiting: (i) a non-enzymatic reaction 1, (ii) the direct GST-like conjugation of GS^–^ to HEDS, and (iii) the formation of Grx-SSEtOH, which might be a reaction intermediate or an inactive dead-end complex. So far, no convincing quantitative model or kinetic law has been reported to explain the enzyme kinetics of the HEDS assay based on a rate-limiting non-enzymatic reaction 1. Although we agree that reaction 1 can affect the overall activity in the assay (Fig. 5), our kinetic data suggest that it is too slow to explain the rapid turnover of HEDS.

Previous analyses usually revealed sequential patterns for the HEDS assay,^2,4,31,32^ and a GST-like conjugation of GS^–^ and HEDS as well as the mechanism suggested by Mieyal *et al.* ^2^ could both explain these patterns. However, depending on the investigated enzyme there are a few exceptions. For example, ping-pong instead of sequential patterns (at least at rather low HEDS concentrations) were described for GSTB1-1 from the bacterium *Proteus mirabilis*. GSTB1-1 is an intermediate between Grx and GST and its cysteine residue at the active site was shown to be essential for catalysis.^33^ Noteworthy, a significantly increased GST-like conjugase activity has also been reported for mutant ScGrx8.^9^ We therefore tested our ScGrx7 preparations for a GST-like conjugase activity using 1 mM GSH, 0.5 mM 1-chloro-2,4-dinitrobenzene and up to 30 μM wild type enzyme, ScGrx7^C108S^ or ScGrx7^K105Y^. ScGrx7^C108S^ served as control to evaluate the potential relevance of the sulfur atom of cysteine 108 and ScGrx7^K105Y^ was intended to mimic the typical exchange of a glutathione binding residue for a catalytic residue in many GST-isoforms.^6^ The detected activities of all three enzymes were identical to negative controls without enzyme. If ScGrx7 has a conjugase activity with these substrates, *v*/[E] would be <10^–4^ s^–1^ (data not shown). Another example for an enzyme with ping-pong patterns in the HEDS assay is wild type ScGrx8,^9^ which has a Trp14-like active site and an altered glutathione-binding site.^8,9^ Mutation of one of both active site cysteines in ScGrx8 abolished the enzymatic activity,^8^ whereas mutation of two non-canonical glutathione-interacting residues drastically increased the enzyme activity and converted the ping-pong patterns to sequential patterns.^9^ Tang *et al.* suggested that the patterns of ScGrx8 are attributed to a higher steady-state concentration of glutathionylated mutant enzyme in the assay so that GSSEtOH becomes depleted and reaction 1 becomes rate-limiting, in particular at low HEDS concentrations.^9^ However, to compensate for the low activity of wild type ScGrx8, such HEDS assays usually contain much higher enzyme concentrations^8,9^ resulting in potentially similar steady-state concentrations of glutathionylated wild type and mutant enzyme. In summary, based on the current knowledge on canonical Grx as well as ScGrx8 and GSTB1-1, the kinetic patterns in the HEDS assay depend on the investigated enzyme or mutant and require a cysteine residue in accordance with a covalent reaction intermediate. At the current stage, a direct Grx-catalyzed conjugation of GS^–^ to HEDS seems to be rather unlikely and the mechanism by Mieyal *et al.* ^2^ is the most simple explanation for the sequential kinetics of ScGrx7.

Substrate or product inhibition can have a significant effect on enzyme kinetic patterns.^34,35^ The GSSEtOH assay revealed that 2-ME is a rather weak product inhibitor of ScGrx7 and allowed us to exclude GSH and GSSG as pattern-altering substrate or product inhibitors for reaction 2 and 3 in Fig. 1. Moreover, the addition of HEDS to the GSSEtOH assay appeared to have no inhibitory effect on ScGrx7 (data not shown) which argues against a HEDS-dependent substrate inhibition with Grx-SSEtOH as an inactive dead-end complex. Based on the assumption that Grx-SSEtOH (instead of Grx-SSG) is also the reaction intermediate in the HEDS assay with ScGrx8, the observed ping-pong/sequential patterns^9^ for wild type/mutant ScGrx8 might be explained by a competitive substrate inhibition of a ping-pong bireactant system in accordance with Segel (p.826–829, Figure IX65)^35^ and our previously proposed ‘glutathione activator model’.^6,8^ In this model, GSH is not only the second substrate of the ping-pong reaction that reduces Grx-SSEtOH but also competes with HEDS for the same binding site. The conversion of ping-pong to sequential patterns then depends on the ratio of the *K*_i_ and *K*_m_ values as well as the substrate concentration.^35^ ScGrx8 mutants with an optimized glutathione activator site should be more susceptible to substrate inhibition by GSH than wild type ScGrx8 and have sequential patterns in accordance with the study by Tang *et al.* ^9^ Furthermore, Lineweaver–Burk plots at variable GSH concentrations should be non-linear and bend up as they approach the *y*-axis in accordance with our previous data.^8^ If the replacement of GSH by HEDS is rather slow, a competition between GSH and HEDS at a glutathione-binding site could also explain the initial lag phase as observed for a variety of Grx when the assay is started by HEDS.^2,4,8^

During the preparation of our manuscript, Mashamaite *et al.* modeled a reversible reaction between PSSG (GSSEtOH) and a reduced dithiol Grx yielding GSH and PSH (2-ME) to explain the sequential kinetic patterns in the HEDS assay.^36^ Even though this model provides an interesting novel twist (with implications for the central question whether Grx also catalyze the glutathionylation of substrates), it does not explain the different kinetic patterns in the HEDS and the GSSEtOH assay, in particular, the lack of convergent lines for the experiments with additional 2-ME in Fig. 3.

What are the physiological implications of our study? The present analysis of the HEDS assay suggests that the detected sequential reaction patterns are not an artifact resulting from a non-enzymatic reaction 1 but actually reflect an alternative Grx-catalyzed reaction pathway with HEDS and GSH as true substrates. As reviewed recently, the activity, mechanism and substrate specificity of Grx is determined by defined reaction geometries and a geometric and electrostatic complementarity between the surfaces of Grx and their substrates.^6,37^ Grx play a key role for the reduction of glutathionylated high and low molecular weight compounds,^7,18,19,38–41^ as well as selected protein disulfide substrates.^5,6,10,28,29^ How these glutathionylated or oxidized proteins are exactly formed often remains to be shown.^19,37,41^ It is also unknown whether small non-glutathione substrates (such as l-cystine, coenzyme A disulfides, or diallyl disulfides and related compounds) are enzymatically or non-enzymatically converted *in vivo*. The outcome of such studies depends on the kinetic competition between high levels of GSH and rather low levels of much more reactive Grx-S^–^ species.^6,37,42^ Our experiments with HEDS were performed with Grx at nanomolar concentrations, suggesting that non-enzymatic reactions might be also outcompeted *in vivo*. However, we used quite high disulfide substrate concentrations. Whether low concentrated disulfides are efficiently converted under physiological conditions should depend on the enzyme/substrate couple because different disulfide substrates such as HEDS and GSSEtOH are apparently turned over in a different and enzyme-specific manner. For example, the *K*appm value of enzyme/substrate couples with ping-pong kinetics decreases when the second substrate concentration is lowered.^34,35^ This allows a high apparent affinity of the enzyme and ensures an efficient turnover under non-saturating conditions. Furthermore, since the true *k*_cat_ and *K*_m_ values of ScGrx7 for GSH and GSSEtOH tended to be infinite, the enzyme cannot be saturated at infinite substrate concentrations, which is in accordance to previous reports on human Grx1 and Grx2,^19,26^ and somehow comparable to many hydroperoxidases.^6,14,43^ In contrast, the *K*appm for enzyme/substrate couples with sequential kinetics remains either constant, as appears to be the case for the ScGrx7/HEDS couple,^4^ or increases, which results in less efficient turnover when the second substrate concentration is lowered. To estimate the relevance of Grx catalysis for disulfide turnover therefore depends not only on the physiological concentration of the substrates but also on the substrate- and enzyme-dependent kinetic patterns.

## Conclusion

We showed that neither substrate depletion nor substrate/product inhibition convert the ping-pong kinetics of ScGrx7 with GSSEtOH to sequential patterns and that the formation of GSSEtOH during the pre-incubation period of the HEDS assay is too slow to account for the high activity of ScGrx7 and other Grx in this standard assay. The sequential patterns of the HEDS assay therefore indicate an alternative Grx mechanism for non-glutathione disulfide substrates in accordance with the ‘glutathione activator model’. Whether Grx and GSH also compete for the reduction of disulfide substrates *in vivo* remains to be addressed in future studies that will have to consider the kinetic patterns for each specific enzyme/substrate couple.

## Experimental section

### Materials

GSH, GSSG, 2-ME and GR from yeast were purchased from Sigma-Aldrich, HEDS was obtained from Alfa Aesar and NADPH was from Gerbu. N-terminally MRGS(H)_6_-tagged ScGrx7 with an altered stop codon^8^ as well as PfGrx, PfGrx^C32S/C88S^ and PfGR from *P*. *falciparum* were expressed in *Escherichia coli* strain XL1-Blue and purified by affinity chromatography as described previously.^4,14,44^ GSSEtOH was synthesized and purified as follows: 230 μL 2-ME (3.28 mmol) were added to a stirred solution of 250 mg GSH (0.81 mmol) in 10 mL H_2_O followed by the dropwise addition of 210 μL H_2_O_2_-solution (30% in water, 2.06 mmol). The mixture was stirred for 1 day at room temperature. The solvent was removed *in vacuo* yielding a colorless oil. After extraction with methanol (5 × 5 mL) and evaporation of the solvent *in vacuo*, a colorless oil was left as residue which was subsequently washed with ice cold ethanol (3 × 2 mL). The product GSSEtOH (198.5 mg, 0.52 mmol, 64%) was obtained as a colorless solid. Further purification was performed by preparative scale RP-18 HPLC (methanol/H_2_O 50 : 50 (v/v), flow rate 12 mL min^–1^, Supelco Ascentis C18, *t*_R_ = 3.75 min). The product was validated by NMR spectrometry and mass spectroscopy: ^1^H-NMR (500.13 MHz, D_2_O): *δ* 2.08–2.13 (m, 2H, CHNH_2_–C*H*_2_–CH_2_), 2.42–2.53 (m, 2H, CH_2_–C*H*_2_–CO), 2.77–2.87 (m, 2H, SS–C*H*_2_–CH_2_OH), 2.88–2.94 (m, 1H, CH–C*H*_2_–SS), 3.16–3.23 (m, 1H, CH–C*H*_2_–SS), 3.75–3.81 (m, 3H, SS–CH_2_–C*H*_2_OH and HOOC–C*H*NH_2_–CH_2_), 3.92 (s, 2H, NH–C*H*_2_–COOH) 4.67–4.70 (m, 1H, NH–C*H*–CH_2_SS). ^13^C-NMR (125.76 MHz, D_2_O): *δ* 26.0, 31.2, 38.7, 39.8, 41.5, 52.7, 53.7, 59.1, 172.7, 173.4 (2C), 174.9. HRMS (ESI^–^): calculated for C_12_H_20_N_3_O_7_S_2_ [M – H]^–^: 382.0748; found: 382.0746.

### GSH:HEDS and GSH:GSSEtOH oxidoreductase assays

Steady-state kinetics of ScGrx7 and PfGrx were determined spectrophotometrically with a thermostated Jasco V-650 UV/vis spectrophotometer by monitoring the consumption of NADPH at 340 nm.^4,8^ All assays were performed at 25 °C in an assay buffer containing 0.1 mM Tris/HCl, 1 mM EDTA, pH 8.0. Before each experiment, stock solutions of 4 mM NADPH, 25 mM GSH, 200 U mL^–1^ GR and 29.4 mM HEDS or 25 mM GSSEtOH were freshly prepared in assay buffer. Final concentrations in the assay were 0.1 mM NADPH, 1 U mL^–1^ GR and 10–20 nM ScGrx7 or PfGrx. GSH was varied between 50 μM and 3.0 mM at fixed concentrations of HEDS (0.18, 0.37 or 0.74 mM) or GSSEtOH (25, 50, 100 or 150 μM). For the HEDS assay, NADPH, GSH and HEDS were pre-incubated for 2 min at 25 °C in order to allow the formation of GSSEtOH before GR was added and a baseline was recorded for 30 s. The assay was then started by the addition of ScGrx7. For the GSSEtOH assay, a baseline of 30 s was recorded after mixing NADPH, GSH, GR, and ScGrx7, and the reaction was initiated by the addition of GSSEtOH. To analyze a potential product inhibition by 2-ME, up to 0.74 mM 2-ME (from a fresh 60 mM stock solution) was added together with GR before the baseline was recorded. The enzyme activity was calculated by subtracting the slope of the baseline and the absorbance of a reference cuvette, which contained all components except for ScGrx7 or PfGrx. *K*appm and *k*appcat were determined by non-linear and linear regression according to Michaelis–Menten, Lineweaver–Burk, Eadie–Hofstee and Hanes theory, using the program SigmaPlot 10.0 (Systat). In addition, a variety of hypothetical equilibrium constants for the reaction between GSSEtOH and 2-ME (ranging from 10^–5^ to 10^6^) was tested to model the concentration of GSSEtOH using eqn (1) with *K* = [HEDS][GSH]/([GSSEtOH][2-ME]). The concentration of free GSH in the assay was calculated by subtracting the concentration of GSSEtOH from the initial GSH concentration.1
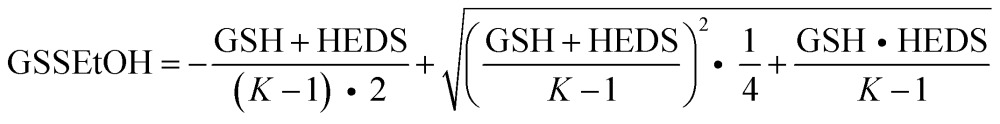



### HPLC and mass spectrometry

The apparent equilibrium constant *K* ^app^ and reaction kinetics of reaction 1 were monitored by HPLC and mass spectrometry. The consumption/formation of HEDS and 2-ME after 2 min incubation of 0.4–4.9 mM GSH and HEDS in assay buffer was monitored at 210 nm by HPLC on a Supelco Ascentis C18 column (5 μm, 250 × 4.6 mm, H_2_O/methanol 75 : 25 (v/v), flow rate 0.8 mL min^–1^). Alternatively, the consumption/formation of GSH, GSSEtOH and GSSG was monitored over time by mass spectrometry after mixing 1.2 mM GSH and 1.2 mM HEDS in assay buffer.

## Author contributions

P.B. and V.S. performed the enzymatic assays. M.D. conceived and supervised the project. The manuscript was written by M.D. and all authors have given approval to the final version of the manuscript.
