# Supplementary material for: Systematic re-evaluation of the bis(2-hydroxyethyl)disulfide (HEDS) assay reveals an alternative mechanism and activity of glutaredoxins
Source: Chem Sci. 2015 May 19;6(7):3788–96. doi: 10.1039/c5sc01051a (PMC5707495; doi:10.1039/c5sc01051a)
Supplement: Supplementary file 1 [file SC-006-C5SC01051A-s001.pdf]

Electronic Supplementary Material (ESI) for Chemical Science.
This journal is © The Royal Society of Chemistry 2015

Supplementary Information

Systematic re-evaluation of the bis(2-hydroxyethyl)disulfide (HEDS) assay reveals an alternative mechanism and activity of glutaredoxins

Patricia Begas, Verena Staudacher, and Marcel Deponte*

Department of Parasitology, Ruprecht-Karls University, D-69120 Heidelberg, Germany

Figure S1:
Alternative evaluation of the HEDS assay kinetics for ScGrx7 (this file)

Figure S2:
Comparison of the HEDS and GSSEtOH assay for PfGrx (this file)

Table S1:
Apparent kinetic parameters of ScGrx7 with GSH and GSSEtOH (this file)

Table S2:
Estimated initial reaction velocities for non-enzymatic reaction 1 (Excel file)

Table S3:
Estimated time-dependent concentration of GSH and GSSEtOH (Excel file)

Table S4:
Comparison of measured reaction velocities in the HEDS and GSSEtOH assay (Excel file)

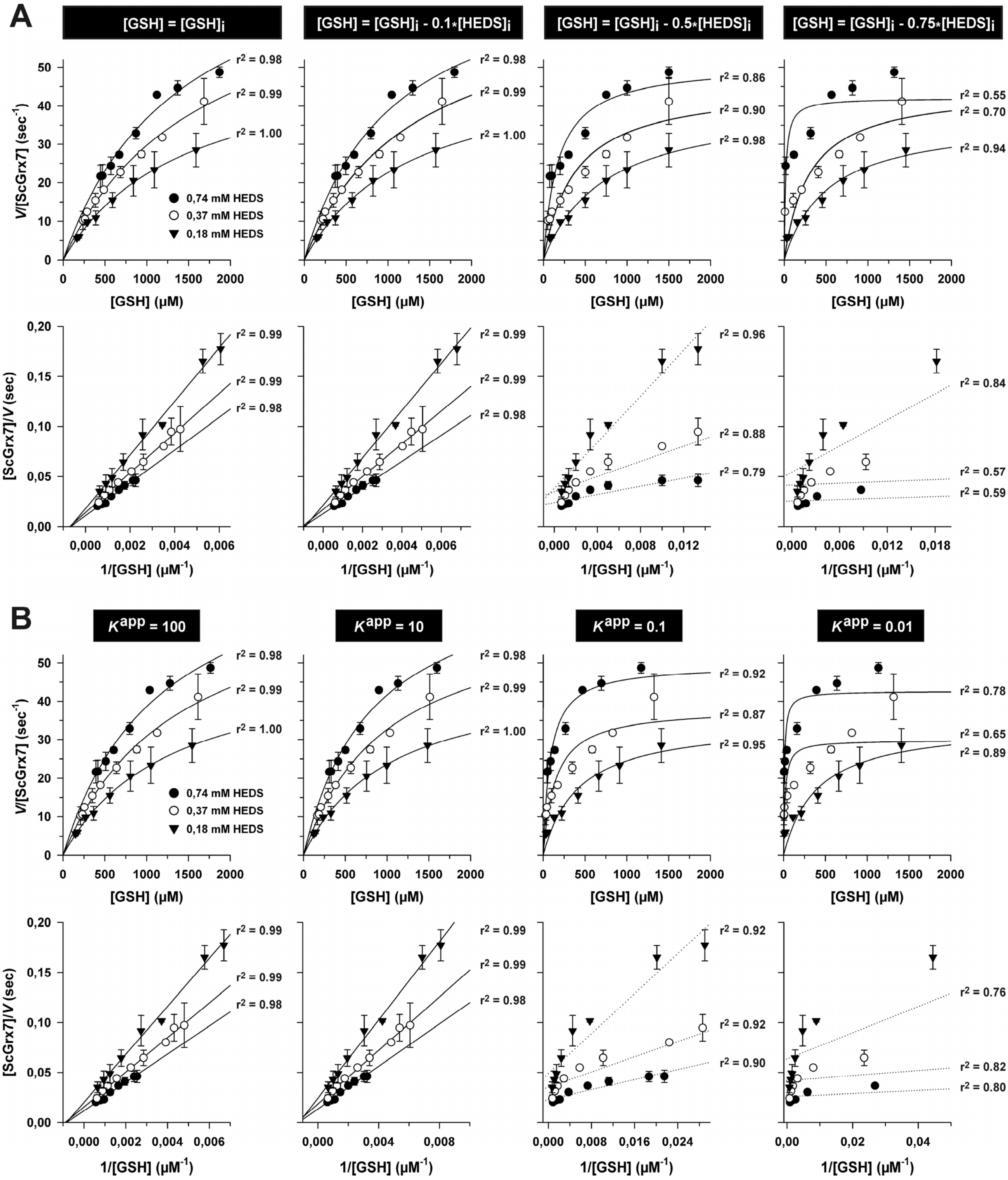

Figure S1. Alternative evaluation of the steady-state kinetics obtained for ScGrx7 in the HEDS assay at three different HEDS and variable GSH concentrations. In both panels, direct and Lineweaver-Burk plots are shown in the upper and lower row, respectively. (A) The concentration of GSH plotted on the x-axis was adjusted assuming a conversion of different amounts of HEDS in reaction 1 before the assay was started by the addition of ScGrx7. $[GSH]_i$ and $[HEDS]_i$ indicate the initial concentrations before pre-incubation. Representative plots for a putative conversion of 0, 10, 50 or 75% HEDS are shown from left to right. (B) The concentration of GSH was calculated using equation 1 based on hypothetical equilibrium constants. Representative plots for K^{app} values of 100, 10, 0.1 or 0.01 are shown from left to right. Values for each data point in panels A and B were averaged from two independent experiments.

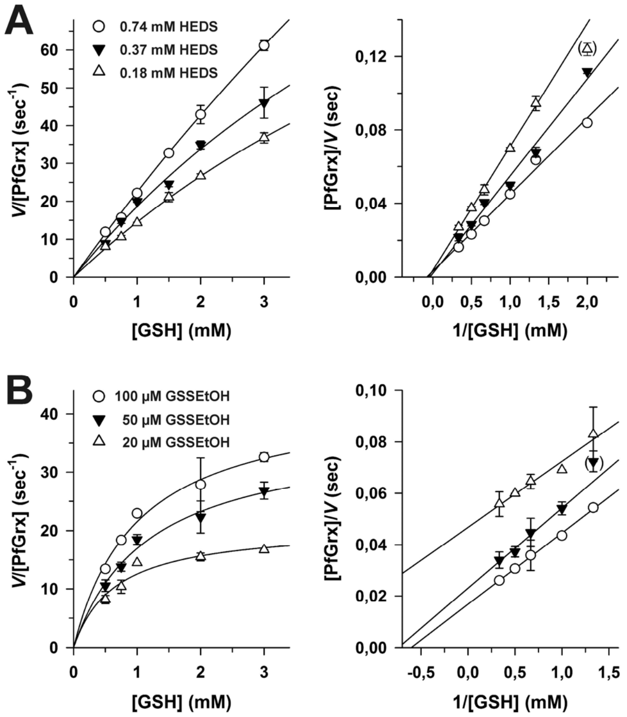

Figure S2. Comparison of the HEDS and GSSEtOH assay for PfGrx. (A) GSH-dependent steady-state kinetics for PfGrx at variable HEDS concentrations. (B) GSH-dependent steady-state kinetics for PfGrx at variable GSSEtOH concentrations. Data points were averaged from replicate measurements of a single protein purification and fitted according to Michaelis-Menten and Lineweaver-Burk theory in the left and right panels, respectively.

Table S1. Apparent kinetic parameters of ScGrx7 with GSH and GSSEtOH.

[GSH] (μM)	[GSSEtOH] (μM)	k_{cat}^{app} (s^{-1})	K_m^{app} (μM)	k_{cat}^{app}/K_m^{app} ($μM^{-1}s^{-1}$)
variable	25	4.7 ± 0.1	11.8 ± 1.0 [a]	0.40 [a]
variable	50	10.2 ± 0.2	33.1 ± 4.9 [a]	0.31 [a]
variable	100	19.4 ± 0.3	53.2 ± 4.6 [a]	0.37 [a]
variable	150	28.0 ± 0.5	62.5 ± 5.1 [a]	0.45 [a]
50	variable	24.2 ± 4.2	136 ± 40 [b]	0.18 [b]
100	variable	37.7 ± 0.9	196 ± 7 [b]	0.19 [b]
200	variable	88.6 ± 18	467 ± 120 [b]	0.19 [b]
300	variable	163 ± 34	853 ± 221 [b]	0.19 [b]
500	variable	244 ± 35	1291 ± 211 [b]	0.19 [b]
1000	variable	708 ± 83	3687 ± 466 [b]	0.19 [b]

[a] $K_m^{app}{}_{(GSH)}$ and $k_{cat}^{app}/K_m^{app}{}_{(GSH)}$
[b] $K_m^{app}{}_{(GSSEtOH)}$ and $k_{cat}^{app}/K_m^{app}{}_{(GSSEtOH)}$
